# Breed-Specific Anaesthetic Mortality in Cats: Evidence from an Analysis of 14,964 Cases

**DOI:** 10.3390/ani16020196

**Published:** 2026-01-09

**Authors:** José I. Redondo, Pablo E. Otero, Fernando Martínez-Taboada, Eva Zoe Hernández-Magaña, Luis Domenech, Jaime Viscasillas

**Affiliations:** 1 Departamento de Medicina y Cirugía Animal, Facultad de Veterinaria, Universidad Cardenal Herrera—CEU, CEU Universities, 46115 Valencia, Spain; eva.hernandezmagana@uchceu.es; 2 Department of Anaesthesiology and Pain Management, Facultad de Ciencias Veterinarias, Universidad de Buenos Aires, Buenos Aires C1427CWN CABA, Argentina; potero@fvet.uba.ar; 3 Escuela de Medicina Veterinaria, Facultad de Ciencias de la Vida, Universidad Andres Bello, Santiago 8370000, Chile; 4 Sydney School of Veterinary Science, University of Sydney, Sydney, NSW 2050, Australia; fer_m_taboada@hotmail.com; 5 Departamento de Matemáticas, Física y Ciencias Tecnológicas, Escuela Superior de Enseñanzas Técnicas, Universidad Cardenal Herrera—CEU, CEU Universities, 46115 Valencia, Spain; luis.domenech@uchceu.es; 6 AniCura Valencia Sur Hospital Veterinario, Picassent 28, 46460 Silla, Spain; jaimeviscasillas2@gmail.com

**Keywords:** cat, anaesthesia, anaesthetic-related mortality, breed-specific risk, brachycephalic cats, peri-anaesthetic death, ASA, multicenter cohort study

## Abstract

Anaesthetic-related mortality in cats is uncommon, yet concerns persist regarding potential breed predispositions and the influence of brachycephalic conformation. This study evaluated breed-specific peri-anaesthetic death before and after adjustment for American Society of Anesthesiologists (ASA) physical status. It explored whether genomic lineage or brachycephalic phenotype was associated with mortality. A prospective, multicentre cohort of general anaesthetics from 198 centres was analysed. Anaesthetic-related death was defined as death during anaesthesia or within 48 h after extubation, excluding euthanasia and deaths attributed to non-anaesthetic causes. Breeds were grouped into four genomic lineages and three brachycephalic phenotypes. Mortality proportions (Wilson 95% confidence intervals) were calculated, and relative risks (RR) were estimated using Poisson regression with robust standard errors, adjusting for ASA class. Among 14,964 cats, 94 deaths occurred (0.63%; 95% CI 0.51–0.77), with mortality increasing from 0.07% (ASA I) to 33.33% (ASA V). After ASA adjustment, most breeds did not differ from European/Domestic Shorthair cats, but Persians remained at increased risk (RR 2.22; 95% CI 1.11–4.46). Mortality did not differ between genomic lineages. Moderate brachycephaly was not associated with an increased risk, whereas brachycephalic breeds (Persian, Exotic Shorthair, Himalayan) showed a higher adjusted risk (RR = 2.33; 95% CI, 1.17–4.63).

## Simple Summary

When cats undergo general anaesthesia for surgery or diagnostic procedures, a small number die during the anaesthetic period or shortly afterwards. Owners and veterinarians often ask whether a cat’s breed, and especially a “flat-faced” head shape, increases this risk. This study analysed anaesthetic records from multiple veterinary centres to estimate the frequency of anaesthetic-related deaths in cats and to compare risk across breeds, while also considering the cats’ pre-anaesthesia illness. Overall, 94 of 14,964 cats died during anaesthesia or within 48 h afterwards, which is about six deaths per 1000 anaesthetics. Deaths were far more frequent in cats that were already severely unwell before anaesthesia. For most breeds, the risk was similar to that of the common European or Domestic Shorthair cat. However, Persian cats remained at a higher risk even after adjusting for pre-anaesthetic health status. “Flat-faced” breeds such as Persian, Exotic Shorthair and Himalayan also showed a higher risk than non-flat-faced cats. These findings are valuable because they help veterinarians communicate risk more clearly to owners, improve planning and monitoring for higher-risk cats, and guide future work on safer anaesthesia for flat-faced breeds.

## 1. Introduction

Anaesthetic-related mortality in cats has decreased over recent decades, coinciding with advances in monitoring, pharmacology, and peri-operative care. Early large-scale studies in the UK reported an anaesthetic-related mortality of 0.24% in cats. They identified strong associations with the American Society of Anesthesiologists (ASA) physical status classification, procedural severity, and extremes of body weight [1]. Later evidence from primary care in the USA confirmed a lower overall mortality rate (0.11%), while still highlighting similar risk factors, showing that increasing age, non-elective procedures, and inadequate pre-anaesthetic assessment significantly raised the odds of death [2]. Even in seemingly healthy cats undergoing routine neutering, peri-anaesthetic complications remain common, most often hypotension, bradycardia, and hypothermia [3]. Recent worldwide data from a large multicentre cohort reported an overall anaesthetic-related mortality of 0.63% in cats, with substantially higher risk in animals with cachexia, higher ASA status, and specific procedures [4]. 

Despite these advances, peri-anaesthetic mortality in cats remains significantly higher than in human medicine, where deaths caused solely by anaesthesia in high-income settings have fallen to just a few dozen per million procedures [5]. In other veterinary patients, mortality remains a key outcome. Recent global analyses in dogs found that anaesthetic-related deaths are similar to those in cats and are significantly influenced by ASA status [6].

Domestic cats encompass diverse breeds with notable differences in morphology, airway conformation, and hereditary disease burden. Genomic analyses have demonstrated distinct genetic structuring among breeds and clustering of variants associated with cardiovascular, metabolic, neuromuscular, and other clinically relevant disorders [7,8]. These findings suggest that inherited traits could influence peri-anaesthetic physiology. Some clinical conditions already show breed associations; for example, inflammatory laryngeal disease appears to be overrepresented in Burmese cats [9]. Brachycephalic breeds such as Persians and Exotic Shorthairs exhibit well-documented upper airway abnormalities, including shortened nasal bones, stenotic nares, aberrant turbinates, and alterations of the nasolacrimal system [10], while morphometric studies indicate that increasing degrees of brachycephaly correlate with worse respiratory scores and louder breathing [11]. 

Evidence from dogs further supports that breed-specific traits can independently influence anaesthetic risk: a recent global analysis revealed that several canine breeds had significantly higher odds of anaesthetic-related death even after adjusting for ASA status and procedural variables [12]. However, it remains uncertain whether cats exhibit similar breed-associated patterns. Earlier feline studies mainly aimed to estimate overall population risk rather than breed differences, and most lacked the statistical strength or detail needed to assess individual breeds [1,2,3,13]. As the global population of pedigreed cats grows and genomic research more often uncovers breed-specific health problems, assessing whether anaesthetic risk differs between breeds has become both urgent and clinically significant.

The primary aim of this study was to quantify breed-specific anaesthetic-related mortality in cats, before and after adjustment for ASA physical status, to distinguish potential breed-associated risk from case-mix differences. Secondary aims were to evaluate mortality within genomic breed clusters described in contemporary feline population genetics work [8], thereby providing biological context rather than replacing breed-level interpretation, and to assess mortality across a three-tier brachycephalic phenotype classification. We hypothesised that: (i) anaesthetic-related mortality would vary across individual breeds (and genomic clusters); (ii) extreme brachycephalic breeds, particularly Persian and closely related breeds, would have higher mortality than non-brachycephalic cats; and (iii) although ASA physical status would remain the dominant predictor, breed- and phenotype-associated differences (if present) would persist, at least in part, after adjustment [8].

## 2. Materials and Methods

This study is a secondary analysis of a prospective, multicentre cohort of feline anaesthetics [4]. Data were collected from 198 veterinary centres across 21 countries in Europe, the Americas, Asia, and Oceania. The most significant contributors were Spain (n = 7483; 50.5%), France (n = 3072; 20.7%), Argentina (n = 2660; 17.9%), the UK (n = 557; 3.8%) and the USA (n = 325; 2.2%).

The parent cohort and data collection procedures were approved by the Ethics Committee of Universidad Cardenal Herrera CEU (CEEA 22/07). This analysis utilised fully de-identified clinical records obtained during routine veterinary care, with no study-specific interventions. All participating centres confirmed adherence to applicable institutional and national regulations regarding data protection and animal welfare. The study adhered to recognised standards for observational epidemiological research [14].

Only cats undergoing general anaesthesia were included; sedation-only procedures were excluded. Peri-anaesthetic information was recorded on a standardised form from premedication through to 48 h post-extubation. The dataset included 14,964 records of cats that had general anaesthesia. The primary endpoint was death within this period. The principal investigator classified deaths into three types: (1) anaesthesia-related, if caused by anaesthesia; (2) euthanasia due to severe injuries; (3) medical or surgical-related, from complications or disease progression. Euthanasia unrelated to anaesthetic issues (n = 98) or non-anaesthetic deaths (n = 21) were excluded. After exclusions, 14,845 cats remained in the database. The outcome was binary: 1 for anaesthetic-related death, 0 for survival. Anaesthetic-related mortality was defined as any anaesthetic-related death occurring during anaesthesia or within 48 h after extubation. For deceased cats, the primary cause was classified from clinical story into six categories—respiratory, cardiovascular, neurological, metabolic, systemic, or other/undetermined—based on clinician reports, not always confirmed by necropsy.

The principal exposure was breed, recorded by the clinician at anaesthesia. Breeds were standardised using international nomenclature. Cats labelled as European, Domestic Shorthair, or similar terms were grouped as European/Domestic Shorthair, the most common category in the dataset and the reference category. Pedigree breeds with enough data were analysed individually.

Genetic lineages were initially classified based on published genomic clustering of domestic cats [8] and tailored to the breeds included in this cohort. Four groups were identified: European Domestic Lineage (comprising European/Domestic and closely related short-haired populations), Ticked Lineage (i.e., Abyssinian, Somali, and Oriental Shorthair), Tabby-patterned Lineage (i.e., Maine Coon, Bengal, Norwegian Forest Cat, Siberian, Toyger, and Highlander), and a Pattern-masked Lineage consisting of the remaining breeds with masked or complex coat patterns.

For each anaesthetic event, the study recorded demographic and clinical data: age, sex, neuter status, weight, ASA status, procedure type, and anaesthetic protocol. ASA status was assigned per the American Society of Anesthesiologists and standardised to a five-level scale (I–V).

The brachycephalic phenotype was coded as a three-level categorical variable (BRACHY). Extreme brachycephalic cats included Persian, Exotic and Himalayan breeds, which correspond to the most severe grades of craniofacial shortening described in CT-based morphological studies [10]. Moderate brachycephalic cats included British Shorthair, British Longhair and Scottish Fold, consistent with intermediate degrees of brachycephaly. All remaining breeds were classified as non-brachycephalic.

### Statistical Analysis

All analyses were performed using R 4.5.2 (R Foundation for Statistical Computing, Vienna, Austria). Categorical variables were summarised as counts and percentages, and continuous variables as medians with interquartile ranges. Mortality proportions for each breed, genetic lineage and brachycephalic category were calculated with 95% confidence intervals (CI) using Wilson’s method.

Crude comparisons used chi-square or Fisher’s exact test for small cell counts. For each breed and lineage, absolute risk differences (ARD) versus the European/Domestic Shorthair were calculated and shown in volcano plots (ARD vs. −log10 *p*-value) to depict effect size and significance. Relative risks (RRs) of anaesthetic-related death were estimated using Poisson regression with a log link and robust (Huber–White) standard errors to account for underestimation of variance with binary outcomes. Adjusted RRs and their 95% CI were presented and displayed using forest plots on a logarithmic scale. Statistical significance was set at *p* < 0.05. Records with missing outcome or covariate data were excluded from the corresponding regression models.

Three analytical frameworks were used. First, breed-level models were fitted for the 10 most common breeds. European/Domestic Shorthair was the reference category, and multivariable models included breed and ASA physical status (I–V) as predictors. Second, anaesthetic-related mortality was compared across the four genetic lineages described above. For these analyses, incidence and crude RRs for each lineage were estimated relative to the European Domestic Lineage. Third, a brachycephalic model was used to evaluate anaesthetic-related mortality across the three brachycephalic categories (non-brachycephalic, moderate brachycephalic, and extreme brachycephalic). In this model, RRs were adjusted for ASA physical status, with the non-brachycephalic group as the reference.

## 3. Results

### 3.1. Anaesthetic-Related Mortality

Out of 14,964 cats, 94 died from anaesthetic causes, yielding a mortality rate of 0.63% (95% CI 0.51–0.77). Mortality increased sharply with ASA class, from 0.07% (95% CI 0.03–0.17; 4/6106) in ASA I and 0.25% (95% CI 0.14–0.42; 13/5297) in ASA II to 0.92% (95% CI 0.64–1.34; 27/2920) in ASA III, 7.01% (95% CI 5.03–9.68; 33/471) in ASA IV and 33.33% (95% CI 21.97–47.03; 17/51) in ASA V. Among anaesthetic-related deaths, respiratory and cardiovascular events were most common. Respiratory causes increased with brachycephaly: 30% in non-brachycephalic cats, 43% in moderately brachycephalic, and 62% in extremely brachycephalic cats. Cardiovascular causes did not conform to this pattern. Other causes of death were less frequent and not linked to the phenotype pattern.

### 3.2. Mortality by Breed

Forty-two breeds were recorded. European/Domestic Shorthair cats (hereafter “European”) formed the reference population (10,007 anaesthetics, 64 deaths; mortality 0.64%, 95% CI 0.50–0.82). Among the 10 most common breeds, crude mortality ranged from 0.00% in several breeds with no observed peri-anaesthetic deaths (e.g., Maine Coon, British Longhair, Sphynx, Burmese) to 1.67% in Persians (8/479; 95% CI, 0.85–3.26) (Table 1).

Compared with Europeans, Persians exhibited a significantly higher mortality rate (*p* = 0.016). Conversely, most other breeds had mortality estimates similar to those of Europeans, although wide confidence intervals were observed for rarer breeds. Breeds with zero observed deaths had upper 95% confidence limits ranging from about 2% to over 40%, indicating considerable statistical uncertainty.

Multivariable Poisson regression with robust variance, adjusted for ASA class, was used to estimate breed-level relative risks of anaesthetic-related death. For breeds with at least one anaesthetic-related death and adequate sample size, most adjusted relative risks did not differ significantly from Europeans (all *p* > 0.05). Persians showed the clearest signal of increased risk, with an adjusted relative risk of 2.22 (95% CI 1.11–4.46; *p* = 0.024). For breeds with very low counts and no deaths, adjusted estimates were numerically unstable and did not provide evidence of either protection or increased risk.

**Figure 1:**
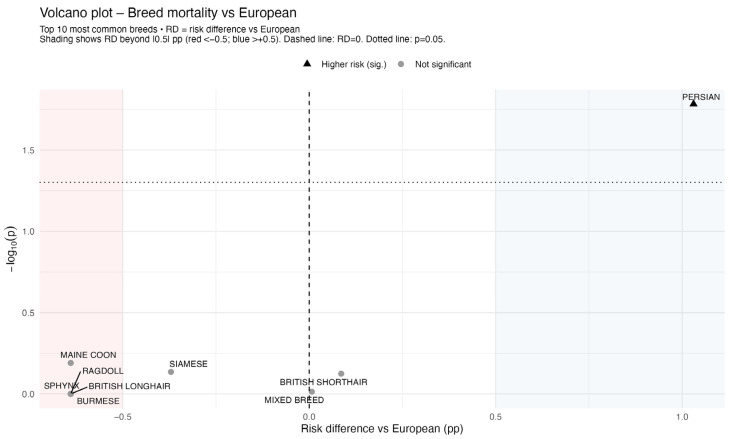
Volcano plot of breed-specific risk differences in anaesthetic-related mortality.

The volcano plot of breed mortality versus Europeans (Figure 1) showed that among the 10 most common breeds, only the Persian breed had a significant difference in risk. Other breeds clustered around the null with minor risk differences and non-significant *p*-values.

### 3.3. Mortality by Genetic Lineage

**Figure 2:**
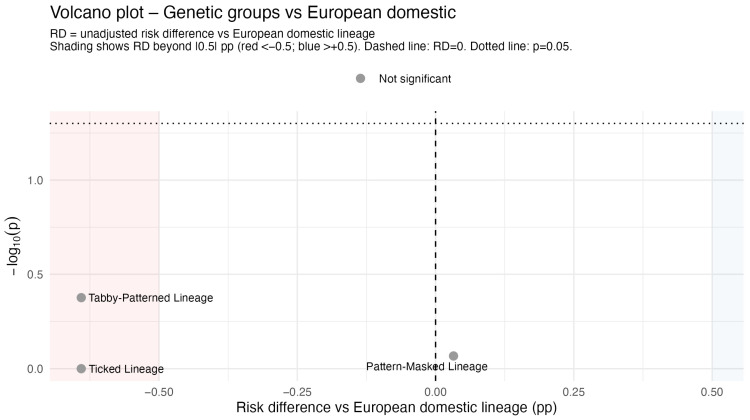
Volcano plot of genetic lineage differences in anaesthetic-related mortality.

Cats were classified by the Lyons genetic lineage (Lyons et al. 2021 [8]), with the European Domestic Lineage as reference (12,175 anaesthetics, 78 deaths; mortality 0.64%, 95% CI 0.51–0.80). Mortality was 0.00% in the Ticked (0/20), 0.00% in the Tabby (0/814), and 0.67% in the Pattern-masked Lineage (11/1633). Fisher’s exact test showed no significant risk differences compared to European (Ticked, *p* = 0.42; Tabby, *p* = 0.42; Pattern-masked, *p* = 0.89). The volcano plot (Figure 2) indicated small risk differences and low −log10(*p*), suggesting no strong lineage effects on anaesthetic mortality, though wide CIs for smaller lineages, especially Ticked, leave moderate differences possible.

### 3.4. Brachycephalic Status

Cats were categorised as non-brachycephalic (n = 14,050), moderately brachycephalic (British Shorthair, British Longhair, Scottish Fold; n = 1152), or extremely brachycephalic (Persian, Exotic Shorthair, Himalayan; n = 303). Mortality in non-brachycephalic cats was 0.58% (82/14,050; 95% CI 0.47–0.73). Moderately brachycephalic cats had a mortality of 0.78% (9/1152; 95% CI 0.41–1.47), and extremely brachycephalic cats 0.99% (3/303; 95% CI 0.34–2.80).

**Figure 3:**
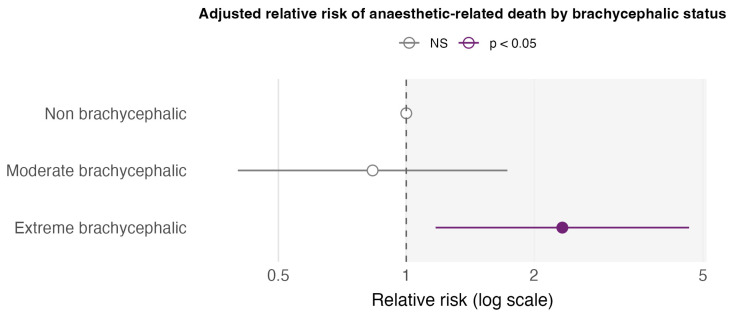
Adjusted relative risk of anaesthetic-related death by brachycephalic status.

In the Poisson regression model adjusted for ASA class, moderately brachycephalic cats did not differ significantly from non-brachycephalic cats (RR 0.83, 95% CI 0.40–1.73; *p* = 0.624). In contrast, extremely brachycephalic cats had an approximately two-fold higher adjusted risk of anaesthetic-related death (RR 2.33, 95% CI 1.17–4.63; *p* = 0.016). The corresponding forest plot (Figure 3) showed a minimal, non-significant risk difference for moderate brachycephaly. In contrast, the extreme brachycephalic category lay within the region indicating both an increased risk and statistical significance relative to the non-brachycephalic reference. Given the small number of deaths in the extreme brachycephalic group, these estimates should nevertheless be interpreted with caution.

## 4. Discussion

In this extensive, prospective, multicentre cohort study of 14,964 feline anaesthetic procedures, the overall anaesthetic-related mortality rate was 0.63%. In the predefined benchmark group of European/Domestic Shorthair cats, mortality was 0.64%. Among common pedigree breeds, crude mortality ranged from 0.00% in Maine Coon and 0.27% in Siamese to 1.67% in Persians. Breeds with zero observed anaesthetic-related deaths should not be interpreted as protected, as limited sample sizes and event counts yield wide confidence intervals and low power, making statistical imprecision a more likely explanation than true biological protection. After adjustment for ASA physical status using robust Poisson models, most between-breed differences were substantially attenuated, with only Persians retaining a statistically significant increased risk compared with European/Domestic Shorthair cats (adjusted relative risk 2.22).

When cats were regrouped by the genomic lineages described by Lyons and colleagues, mortality in the European Domestic, Tabby-patterned, Ticked and Pattern-masked lineages was very similar, and none differed significantly from the European Domestic reference [8]. This mirrors the limited discriminatory value of broad groupings observed in dogs, in which a worldwide analysis revealed substantial heterogeneity at the breed level despite apparently homogeneous clusters [12]. Collectively, these findings suggest that ancestral or coat-pattern categories are too coarse to capture the conformational or pathophysiological traits that determine peri-anaesthetic risk and, from a clinical perspective, support concentrating mitigation efforts on recognisable extreme phenotypes rather than on abstract genomic cluster membership.

Stratification by brachycephalic phenotype provided a more evident, clinically interpretable contrast. Moderately brachycephalic breeds (British Shorthair, British Longhair, Scottish Fold) had mortality estimates indistinguishable from those of non-brachycephalic cats after adjustment for ASA physical status. By contrast, the extremely brachycephalic group (Persian, Exotic Shorthair, Himalayan) showed an approximately twofold higher adjusted risk than non-brachycephalic cats. In combination with the breed-level analysis, this indicates that the excess mortality signal is concentrated in extreme brachycephalic phenotypes rather than reflecting a generic feature of pedigree status or mild brachycephaly.

Cause-of-death patterns further support a phenotype-specific interpretation. When the presumed primary mechanism of death was stratified by brachycephalic phenotype, respiratory complications were more frequent in brachycephalic categories: respiratory causes accounted for 20/67 (30%) deaths in non-brachycephalic cats, 6/14 (43%) in moderately brachycephalic cats and 8/13 (62%) in extremely brachycephalic cats. Cardiovascular events did not exhibit a comparable gradient, suggesting that the phenotype signal is not consistently elevated across all peri-anaesthetic causes. These mechanistic inferences should be interpreted cautiously because causes were derived from case narratives and were not uniformly confirmed by necropsy; nevertheless, they identify phenotype-informed priorities for risk mitigation, particularly airway management and close monitoring during early recovery.

The biological plausibility of this pattern is supported by prior work linking feline brachycephaly to pronounced alterations in upper airway anatomy, including foreshortened nasal bones, stenotic nares and aberrant turbinates, which increase airway resistance and may reduce respiratory reserve during anaesthesia [10]. Owner-reported and morphometric data similarly associate increasing degrees of brachycephaly with higher respiratory scores and more pronounced breathing noise [11]. In addition, retrospective multicentre studies highlight laryngeal disease as an important, and likely under-recognised, contributor to respiratory morbidity in cats [9]. It is therefore credible that, even after accounting for general health status via ASA, Persian and related extreme brachycephalic cats retain an anatomy-driven susceptibility to airway compromise that is not shared by other breeds.

In the broader context of feline anaesthetic risk, the overall mortality of 0.63% lies at the higher end of recent reports but remains consistent with contemporary estimates [1,2,3,15,16]. The higher figure observed here is most likely attributable to differences in case mix, study design, and outcome definition, rather than to an actual temporal increase in anaesthetic risk. A comprehensive explanation of these differences is provided in the parent study [4]. Procedure-specific studies focusing on low-risk, elective neutering in healthy cats have reported substantially lower peri-anaesthetic mortality, typically below 0.1%, likely reflecting the selection of ASA I–II patients and more standardised protocols [16]. The present study encompasses a broader spectrum of case severity, urgency and invasiveness, which may partly explain the higher global estimate.

Consistent with previous work, mortality increased steeply with ASA class, confirming ASA physical status as the dominant predictor of outcome in feline anaesthesia and reinforcing the importance of case severity, urgency and procedural invasiveness in explaining risk variation [1,2,4]. Within this ASA-centred framework, the persistent Persian signal after adjustment should be viewed as a specific exception rather than a competing explanatory model.

Clinically, these results suggest that breed alone should rarely drive decision-making. For most common breeds, absolute risk was low and closely aligned with that of European/Domestic Shorthair cats, supporting a patient-focused approach to risk communication in which owners are informed that systemic health and procedure type are the principal determinants of outcome. The clear exception is the group of extremely brachycephalic cats, for which enhanced airway planning appears warranted. In Persians and related breeds, clinicians may consider strategies such as experienced intubation, contingency planning for a difficult airway [17], cautious titration of sedatives, proactive ventilatory and thermal support, and prolonged, closely supervised recovery to mitigate anatomy-driven vulnerability not fully captured by ASA grading.

Several limitations warrant emphasis. The multivariable models adjusted for ASA physical status only; although ASA is a significant determinant of peri-anaesthetic mortality, it does not capture procedure urgency, surgical invasiveness, duration of anaesthesia, airway management strategy or breed-associated comorbidities, so residual confounding is likely and associations should be interpreted as risk markers rather than evidence of causality. Event counts were small across many breeds and lineage categories, necessitating restricting adjusted models to the most frequent breeds and yielding wide confidence intervals for some estimates. Breed and brachycephalic status were assigned phenotypically rather than by genetic testing or formal morphometric assessment, so non-differential misclassification is likely and would tend to attenuate true associations. The coat pattern was not recorded at the case level; for breeds spanning several coat-pattern categories in the Lyons classification, individuals were pragmatically assigned to the lineage corresponding to the most prevalent pattern for that breed, which may have misclassified some cats and biased lineage comparisons towards the null [8]. Attribution of cause and anaesthetic relatedness may vary across centres and practice types. If misclassification is largely non-differential, relative risks would be biased towards the null; if differential, for example, if clinicians have a lower threshold to attribute deaths to anaesthesia in brachycephalic cats, contrasts could be inflated or attenuated unpredictably. Detailed data on comorbidities such as cardiomyopathy or subclinical airway disease, and complications beyond 48 h, were not available. The observation period extended from premedication to 48 h after extubation, whereas other studies used longer follow-up windows, in some cases up to 7 days [2,18]. Follow-up duration should therefore be considered when comparing mortality rates across studies, because shorter windows may miss late events and longer windows may capture deaths less directly attributable to anaesthesia. Finally, moderate agreement among experts in classifying peri-anaesthetic deaths has been demonstrated experimentally, indicating that outcome attribution itself can be a source of variability that affects comparability [19].

The dataset integrates anaesthetic procedures from a large number of veterinary centres operating under diverse clinical protocols. This heterogeneity increases generalisability and reduces the influence of idiosyncratic practice styles. Centre-level modelling was not pursued because the aim was not to compare hospitals but to obtain population-level estimates across routine clinical settings. Future work could evaluate specific practices that may influence risk in vulnerable phenotypes, including the use of supraglottic airway devices, induction techniques, peri-anaesthetic ventilatory strategies, and recovery-room monitoring, particularly in Persian and other extreme brachycephalic cats, in which airway instability may play a key mechanistic role. Potential repeated anaesthetics in the same individual could not be reliably identified and therefore could not be explicitly modelled.

## 5. Conclusions

Overall, this global breed-specific analysis indicates that anaesthetic-related mortality in cats remains significant and is heavily influenced by ASA physical status. Most breeds exhibit a risk profile similar to that of European/Domestic Shorthair cats. Conversely, Persian and closely related extreme brachycephalic breeds seem to carry an inherent, anatomy-driven increased risk. Future research should aim to more precisely define the anatomical and physiological mechanisms underlying this vulnerability, document the peri-anaesthetic course in Persian and related cats in greater detail, and evaluate targeted peri-anaesthetic strategies to enhance safety in these animals, all while maintaining an ASA-centred framework for the broader feline population.

## Figures and Tables

**Table 1:** Breed-specific mortality related to anaesthesia in cats across the 15 most common breeds.

Breed	Total	Deaths	Mortality % (95% CI)	*p* (Crude)	RR (95% CI)	*p* (adj)
European/Domestic Shorthair	10,007	64	0.64 (0.50–0.82)	REF	REF	REF
Mixed Breed	2165	14	0.65 (0.39–1.08)	0.970	1.09 (0.62–1.90)	0.764
British Shorthair	965	7	0.73 (0.35–1.49)	0.751	0.89 (0.43–1.84)	0.754
Persian	479	8	1.67 (0.85–3.26)	0.016	2.22 (1.11–4.46)	0.024
Siamese	372	1	0.27 (0.05–1.51)	0.731	0.28 (0.04–2.10)	0.215
Maine Coon	211	0	0.00 (0.00–1.79)	0.644	NE	NE
British Longhair	85	0	0.00 (0.00–4.32)	1.000	NE	NE
Sphynx	84	0	0.00 (0.00–4.37)	1.000	NE	NE
Burmese	80	0	0.00 (0.00–4.58)	1.000	NE	NE
Ragdoll	64	0	0.00 (0.00–5.66)	1.000	NE	NE
Sacred Birman	50	0	0.00 (0.00–7.13)	1.000	NE	NE
Russian Blue	46	0	0.00 (0.00–7.71)	1.000	NE	NE
Norwegian Forest Cat	39	0	0.00 (0.00–8.97)	1.000	NE	NE
Exotic	31	0	0.00 (0.00–11.03)	1.000	NE	NE
Turkish Angora	28	0	0.00 (0.00–12.06)	1.000	NE	NE

REF: Reference. NE: Not estimable. Breeds with zero peri-anaesthetic deaths did not allow estimation of an adjusted relative risk (RR) in the Poisson models because of the absence of events (structural zeros). For these breeds, adjusted RRs are reported as NE (not estimable); any model-derived numerical values (for example, RR close to 0 with very narrow confidence intervals) represent statistical artefacts and should not be interpreted as evidence of a protective effect.

## Data Availability

The data supporting the findings of this study are available from the corresponding author upon reasonable request.

## Abbreviations

The following abbreviations are used in this manuscript:

ASA	American Society of Anesthesiologists physical status classification
CEEA	Comité de Ética en Experimentación Animal (Ethics Committee)
CIs	Confidence intervals
RR	Relative risk
